# Blood-feeding patterns of *Anopheles *mosquitoes in a malaria-endemic area of Bangladesh

**DOI:** 10.1186/1756-3305-5-39

**Published:** 2012-02-15

**Authors:** Kabirul Bashar, Nobuko Tuno, Touhid Uddin Ahmed, Abdul Jabber Howlader

**Affiliations:** 1 Laboratory of Entomology, Department of Zoology, Jahangirnagar University, Savar, Dhaka 1342, Bangladesh; 2 Laboratory of Ecology, Faculty of Natural Science and Technology, Kanazawa University, Kanazawa, Japan; 3 Institute of Epidemiology, Disease Control & Research (IEDCR), Mohakhali, Dhaka-1212, Bangladesh

## Abstract

**Background:**

Blood-feeding patterns of mosquitoes are crucial for incriminating malaria vectors. However, little information is available on the host preferences of *Anopheles *mosquitoes in Bangladesh. Therefore, the objective of the present study was to determine the hematophagic tendencies of the anophelines inhabiting a malaria-endemic area of Bangladesh.

**Methods:**

Adult *Anopheles *mosquitoes were collected using light traps (LTs), pyrethrum spray (PS), and human bait (HB) from a malaria-endemic village (Kumari, Bandarban, Bangladesh) during the peak months of malaria transmission (August-September). Enzyme-linked immunosorbent assay (ELISA) and polymerase chain reaction (PCR) were performed to identify the host blood meals of *Anopheles *mosquitoes.

**Results:**

In total, 2456 female anopheline mosquitoes representing 21 species were collected from the study area. *Anopheles vagus *Doenitz (35.71%) was the dominant species followed by *An. philippinensis *Ludlow (26.67%) and *An. minimus *s.l. Theobald (5.78%). All species were collected by LTs set indoors (n = 1094), 19 species were from outdoors (n = 784), whereas, six by PS (n = 549) and four species by HB (n = 29). Anopheline species composition significantly differed between every possible combination of the three collection methods (χ^2 ^test, P < 0.001). Host blood meals were successfully detected from 1318 (53.66%) *Anopheles *samples belonging to 17 species. Values of the human blood index (HBI) of anophelines collected from indoors and outdoors were 6.96% and 11.73%, respectively. The highest values of HBI were found in *An. baimai *Baimaii (80%), followed by *An. minimus *s.l. (43.64%) and *An. annularis *Van den Wulp (37.50%). *Anopheles baimai *(*B_i _*= 0.63) and *An. minimus *s.l. (*B_i _*= 0.24) showed strong relative preferences (*B_i_*) for humans among all hosts (human, bovine, goats/sheep, and others). *Anopheles annularis*, *An. maculatus *s.l. Theobald, and *An. pallidus *Theobald exhibited opportunistic blood-feeding behavior, in that they fed on either humans or animals, depending on whichever was accessible. The remaining 12 species preferred bovines as hosts.

**Conclusions:**

The observed high anthropophilic nature of *An. baimai*, *An. minimus *s.l., and *An. annularis *revealed these species to be important malaria vectors in hilly areas of Bangladesh. Higher values of HBI in outdoor-resting mosquitoes indicated that indoor collection alone is not adequate for evaluating malaria transmission in the area.

## Background

**Figure 1:**
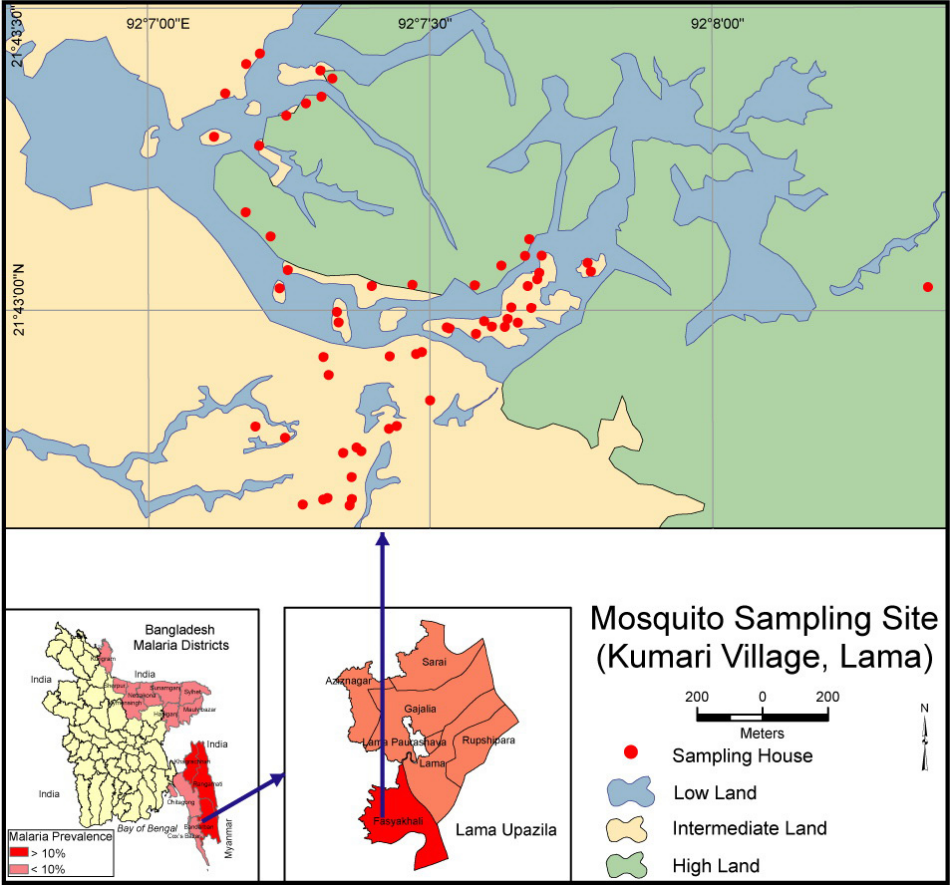
**Sampling area in Kumari, Lama, Bangladesh**. Low lands = rice field or water bodies, Intermediate lands = foothills, High lands = hilly forest.

Malaria is endemic to Bangladesh (formerly Bengal), and due to the efforts of the nation's Malaria Eradication Program, malaria is now confined to the northeastern border areas of Bangladesh. Thirteen of 64 districts bordering India and Myanmar are severely affected by malaria [1]. Among these, the districts of Chittagong, Rangamati, Khagrachari, Bandarban, and Cox's Bazar are hyperendemic, whereas the districts of Kurigram, Sherpur, Mymensingh, Netrakona, Sylhet, Sunamgonj, Moulvibazar, and Hobiganj are prone to low-level epidemics. In 2009, 47 deaths with 63,871 laboratory-confirmed cases and 553,787 clinical cases were reported [2]. Most infections are caused by *Plasmodium falciparum *in all districts except for Kurigram, where *P. vivax *(75.07%) is dominant [3]. The highest incidence of *P. falciparum *(93.16%) in Bangladesh has been reported from the three hilly districts (Bandarban, Khagrachari, and Rangamati) [4]. The average malaria prevalence in these three districts is 11.7% (Figure 1) [1], and these areas remain highly conducive to malaria due to uncontrolled immigration, political unrest, and hilly terrain.

Seven anopheline species have been incriminated as competent malaria vectors in Bangladesh [5]. Four of these seven species (*Anopheles baimai *Baimaii, formerly known as *An. dirus *species D; *An. philippinensis *Ludlow; *An. sundaicus *Rodenwaldt; and *An. minimus *s.l. Theobald) have been considered the primary vectors [6]. In hilly districts, the principal malaria vectors are *An. baimai *and *An. minimus *s.l., whereas *An. philippinensis *and *An. minimus *s.l. dominate in flat plane areas of Bangladesh [6]. The other three species, *An. aconitus *Doenitz, *An. annularis *Van der Wulp, and *An. vagus *Doenitz, transmit malaria during outbreak situations [7-10].

Anophelines exhibit a wide range of host preferences such as humans, livestock, birds, and reptiles [11-13], and the prevalence of malaria is influenced by mosquito host selection [14]. If mosquitoes do not discriminate among host species, the proportion of blood meals attributable to specific hosts would reflect the relative abundance of host animals. Alternatively, certain mammalian species might be more attractive or accessible for specific mosquito species. Such host preferences, especially the degree of anthropophily, would affect the efficacy of the malaria vector. Climatic, environmental, and socioeconomic factors also influence vector populations by determining feeding behavior and vectorial capacity of malaria transmission [15-17]. Understanding the blood-foraging patterns of insects in the field is tantamount to implicating vectors [18]. However, little information is available regarding the host preferences and blood-feeding behavior of *Anopheles *species in Bangladesh. Therefore, the present study was conducted to document the hematophagic tendencies of confirmed and suspected malaria vectors in a malaria-endemic area of Bangladesh.

## Methods

### Study area

The study was conducted in the isolated (closest village is 2 km away), highly malaria-prone village of Kumari (21°46'30"N, 92°12'00"E; 26-72 m above sea level) in Lama Upazila, Bandarban District, Bangladesh (Figure 1). The study area is located in a tropical monsoon-type climate zone, with hot and rainy summers and a pronounced dry season during cooler months. The climate is one of the wettest in the world, with a monthly average rainfall of more than 317.8 mm [19]. Major portions of the study area are vegetated by secondary forest with interspersed rubber plantations. Mosquito breeding sites within the village include a narrow, slow-running stream and many wells, pools, ponds, and rice fields. The village of Lama resides within several hillsides and is inhabited by 1218 individuals (male percentage: 53.94%) in 137 houses. Most families in the village keep livestock in their compound, including bovines, goats, and sheep. A typical compound consists of a human residence and a shed for animals. The majority of houses are made of mud walls with a tin roof and an opening between the wall and the roof for aeration. Doors and windows are normally kept open until people go to bed. The study village was selected from among several applicant villages because of its high incidence of malaria and because it serves as a typical village in the hilly district, based on its landscape composed of scattered houses, secondary forest, and rubber plantations. We conducted preliminary sampling of malaria vector species within several villages from April to June 2010, after which we focused on Lama, where most of the malaria vector species could be sampled in one village.

### Mosquito collections

The period from May to August has been reported as the peak malaria season in Bangladesh [20]. Mosquitoes were collected from 28^th ^August to 8^th ^September 2010, both indoors and outdoors in 58 selected houses from the study area using light traps (LTs), pyrethrum spray (PS), and human bait (HB) following World Health Organization procedures [21]. Ethical clearance was obtained from Jahangirnagar University, the head of the village, and the office of the Faisha Khali Union Parishod (local government), Lama Upazila, Bandarban. Collectors and individuals serving as human bait were given antimalarial drugs for disease prevention.

### Mosquito identification

Collected mosquitoes were kept in paper cups and brought to the field laboratory for identification. At the laboratory, mosquitoes were anesthetized with chloroform and morphologically identified under stereomicroscopes, within 24 h after sampling using taxonomic keys [22-26]. After field sampling, mosquitoes were transported to the laboratory at Jahangirnagar University and frozen. All samples were then transported to the ecology laboratory of Kanazawa University for verifying species and blood-meal identification.

### Sample preparation and blood-meal analysis

Collected females *Anopheles *were prepared for a blood meal test. The abdomen of the mosquitoes was separated from the head and thorax and homogenized in 250 μl of phosphate-buffered saline (PBS; pH 7.4). Anophelines were tested for blood meals of humans, bovines, and goats using enzyme-linked immunosorbent assays (ELISAs) as described by Beier *et al. *[27] with slight modification. All abdomens were soaked in 250 μl of PBS before the test, and 50 μl was then added to each well of three types (bovine, goat/sheep, human) of ELISA plates, with two replicates per type. The samples were visually judged by comparison with negative and positive samples. If three of three trials were positive, we judged the sample as positive. We also applied molecular methods to samples judged as negative in ELISA. DNA was extracted from residual mosquito abdomens. Briefly, mosquitoes were homogenized in 120 μl of warm DNA extraction buffer (DEB: sodium dodecyl sulfate (SDS), 0.5M EDTA, 5M NaCl, 1.0M Tris buffer (pH 8)) and 1.5 μl of RNAse A was added. Then 3 μl of proteinase K was added to each homogenate and incubated at 50°C for 60 minutes. After incubation 60 μl of each phenol and chloroform were added and centrifuged for 10 minutes. The supernatant was transferred to a tube and 300 μl of ice-cold 95% ethanol was added and allowed to precipitate overnight in the -20°C freezer. Samples were then centrifuged for 10 minutes at the highest speed (13000 rpm) and the supernatant was discarded, 300 μl of ice-cold 70% ethanol was then added without disturbing the pellet. Finally, the supernatant was discarded and dried pellets were resuspended in 100 μl of TE pH 7.4. Extracted DNA was diluted in double distilled water at a 1:1000 dilution. PCR was conducted using primers of humans, bovines, dogs, goats/sheep, and pigs as described by Kent and Norris [28]. One microliter of extracted DNA from a single mosquito abdomen was applied as a DNA template for a reaction of 20 μl using PCR mastermix (GoTaq; Promega KK, Tokyo, Japan). Along with positive and negative controls, 10 μl of amplified product was used for electrophoresis in a 2% agarose gel. To identify the host blood meals, the size of the amplified products was measured under UV lights by comparison with DNA ladder markers. When no DNA was amplified, nested PCR was applied twice using the previous PCR products as the DNA template.

### Host census

We visited all sampling houses to collect information about cases of malaria, the number of family members, and available mammal hosts kept in the compound using standard pretested questionnaires. We used host data to calculate the forage ratio and selection index (see below). Epidemiological information was used for sampling mosquitoes but was not directly applied in the data analysis.

### Data analysis

We compared species composition among the three sampling methods and evaluated the bias in respective species using chi-square (χ**^2^**) tests with Bonferroni correction. To demonstrate the anthropophilic nature of *Anopheles *mosquitoes, the proportion of human blood within an entire mosquito blood meal was calculated as the human blood index (HBI). We also calculated the forage ratio (*w_i_*), [29,30] and selection index (*B_i_*) of Manly *et al. *[31] to quantify host preferences among humans, bovines, goats/sheep, and others. The forage ratio *w_i _*for species *i *was calculated as

(1) $$w_{i}=\frac{o_{i}}{p_{i}}$$

where *w_i _*is the forage ratio for mosquito species *i*, *o_i _*is the proportion of host species *i *in the blood meals, and *p_i _*is the proportion of host species *i *available in the environment.

The selection index for species *i *was calculated as

(2) $$B_{i}=\frac{w_{i}}{\sum_{i=1}^{n}w_{i}}$$

Where *B_i _*is the selection index for mosquito species *i*, *w_i _*is the forage ratio for mosquito species *i*, and *n *is the number of different types of blood sources available.

Dogs were not considered in the calculations of *w_i _*and *B_i _*because PCR identified only one dog blood meal among all samples tested. Forage ratios were calculated in two ways: using values of *o_i _*and *p_i _*from direct counts of animals and using log-transformed data [log (*p_i_*+1)] to take into account the fact that animals are not distributed at random in the study area. With *n *types of host species, values of 1/*n *of the standardized forage ratio or the selection index (*B_i_*) indicate no preference, those below 1/*n *indicate relative avoidance, and values above 1/*n *indicate relative preference of host species *i*. G-tests [31] were conducted to compare these values to the null hypothesis that mosquitoes have no preference for particular hosts. Standard errors and confidence limits were calculated to determine the statistical significance of the forage ratio (*w_i_*) and selection index (*B_i_*). Bonferroni correction [31] was applied for multiple comparisons.

The geographical positions of the sampling houses were determined using handheld GPS (Garmin Oregon 550). ArcView GIS 3.3 and Arc GIS 9.2 software were used for map preparation of the sampling area.

## Results

### Species composition

In total, 2456 female anopheline mosquitoes belonging to 21 species were collected using light traps (LTs), pyrethrum spray (PS), and human bait (HB) (Table 1). All 21 species were collected using LTs set indoors (n = 1094), and 19 species were collected with outdoor LTs (n = 784). In contrast, only six and four species were collected using PS (n = 549) and HB (n = 29), respectively (Table 1). Mosquito species composition significantly differed between every possible combination of the three collection methods (χ**^2 ^**test, P < 0.001). The catches of LT, PS, and HB methods were compared for the dominant nine mosquito species (sample sizes > 50). *Anopheles vagus *and *An. subpictus *Grassi were collected more often by PS (χ**^2 ^**test, P < 0.001), whereas the seven species (*An. vagus, An. philippinensis*, *An. minimus *s.l., *An. peditaeniatus *Leicester, *An. barbirostris *Van den Wulp, *An. karwari *James and *An. umbrosus *Theobald) were collected more often by LTs (χ**^2 ^**test, P < 0.05). Comparisons between LTs and HB indicated that *An. philippinensis *and *An. minimus *s.l. were collected more often in HB (χ**^2 ^**test, P < 0.05), whereas five species (*An. vagus*, *An. peditaeniatus*, *An. barbirostris*, *An. karwari *and *An. umbrosus*) were collected more frequently with LTs (χ**^2 ^**test, P < 0.05); catches of two species did not significantly differ between the two methods. For comparisons between HB and PS, *An. philippinensis *and *An. minimus *s.l. were collected more often with HB (χ**^2 ^**test, P < 0.001), whereas the other six species (*An. vagus, An. subpictus*, *An. peditaeniatus*, *An. barbirostris*, *An. aitkenii *and *An. jamesii*) were collected more often with PS (χ**^2 ^**test, P < 0.01). Mosquito species composition significantly differed between indoor and outdoor LT collections (χ**^2 ^**test, P < 0.001). For the dominant nine (*An. vagus*, *An. philippinensis*, *An. minimus *s.l. *An. peditaeniatus*, *An. barbirostris*, *An. karwari*, *An. umbrosus, An. hyrcanus *group and *An. nigerrimus*) species (sample sizes were more than 50 for LT catches), *An. peditaeniatus *and *An. nigerrimus *Giles were captured more often via indoor LTs, whereas *An. umbrosus *was caught more frequently in outdoor LTs (χ**^2 ^**test, P < 0.001; Table 1).

**Table 1:** *Anopheles *species composition as the proportion collected using light traps (LTs), pyrethrum spray (PS), and human bait (HB) in Kumari, Bangladesh, from August to September 2010

	Method	LT			PS	HB			
				
Species		Indoors	Outdoors	χ2*			χ2		
								
	N = 2456	N = 1094	N = 784	In vs. Out	N = 549	N = 29	LT vs. PS	LT vs. HB	PS vs. HB
*An. vagus*	879	0.182	0.212	ns	0.936		< 0.001	< 0.001	< 0.001
*An. philippinensis*	655	0.338	0.335	ns		0.759	< 0.001	< 0.001	< 0.001
*An. minimus *s.l.	142	0.081	0.065	ns		0.069	< 0.05	0.022	< 0.001
*An. peditaeniatus*	139	0.094	0.045	< 0.001	0.002		< 0.001	0.009	< 0.001
*An. barbirostris*	130	0.059	0.083	ns	0.002		< 0.001	0.014	< 0.001
*An. karwari*	128	0.073	0.061	ns			< 0.05	0.011	< 0.001
*An. umbrosus*	128	0.032	0.119	< 0.001			< 0.05	0.011	< 0.001
*An. hyrcanus *group	52	0.031	0.023	ns			ns	ns	0.004
*An. nigerrimus*	52	0.045	0.004	< 0.001			ns	ns	0.004
*An. subpictus*	40	0.005	0.005		0.056				
*An. pallidus*	24	0.011	0.015						
*An. baimai*	23	0.015	0.005			0.103			
*An. maculatus *s.l.	22	0.015	0.008						
*An. annularis*	18	0.005	0.013			0.069			
*An. jeyporiensis*	7	0.005	0.003						
*An. kochi*	5	0.003	0.003						
*An. aitkenii*	4	0.002	0.001		0.002				
*An. jamesii*	3	0.001	0.001		0.002				
*An. tessellatus*	2	0.002							
*An. varuna*	2	0.002							
*An. fluviatiles*	1	0.001							

Total		1	1		1	1			

*χ2 tests were applied for the dominant species (n > 50) to compare the proportions of indoor vs outdoor LT catches as well as LT vs. PS, PS vs. HB, and HB vs. LT; ns = not significant.

### Feeding status and blood-meal host identification

Mosquito feeding status was visually classified as unfed (UF), fed (F), half-gravid (HG), or gravid (G). The highest percentage of specimens was UF (49.59%), followed by F (30.46%), HG (17.59%), and G (2.36%). We were able to successfully identify the host animals of the majority of F (96.79%) and HG (95.60%) mosquito blood meals. A sizable percentage (10.67%) of blood meals visually categorized as UF reacted as blood-meal positive. The host blood meals of 50 engorged (F, HG, and G) mosquitoes were not identified (Table 2).

**Table 2:** Number of blood meals identified and their feeding status

Feeding status	Number of individuals (%)	Blood Meal Identified (%)	BM not identified (%)
Blood-fed	748 (30.46)	724 (96.79)	24 (3.21)
Gravid	58 (2.36)	51 (87.93)	7 (12.07)
Half-gravid	432 (17.59)	413 (95.60)	19 (4.40)
Unfed	1218 (49.59)	130 (10.67)	

Total	2456 (100.00)	1318 (53.66)	50 (3.65)

### Potential host composition

During the sampling period, we observed 403 humans, 156 bovines, 98 goats and sheep, and 36 dogs in the sampled houses.

### Blood feeding of anophelines collected indoors and outdoors

Seventeen *Anopheles *species of 21 tested positive for blood meals. The total numbers of tested blood meals from indoor and outdoor collections were 1647 and 809, respectively. Of 1318 mosquito blood meals, 977 (74.13%) were collected indoors, and the remainder was collected outdoors (Table 3). The highest human blood index (HBI) was found in *An. baimai *(in: 66.7%, n = 3; out: 100%, n = 2), followed by *An. minimus *s.l. (in: 47.06%, n = 34; out: 38.1%, n = 21), *An. annularis *(in: 50%, n = 4; out: 33.33%, n = 3), and *An. pallidus *Theobald (in: 37.5%, n = 8; out: 50%, n = 4). In five taxa (*An. hyrcanus *group, *An. jamesii *Theobald, *An. maculatus *s.l. James, *An. peditaeniatus*, and *An. subpictus*), human blood was not detected outdoors but was detected in indoor collections. Lone species (*An. umbrosus*) collected from indoors found 0% HBI. The highest HBI was observed in *An. baimai *(80%, n = 5), followed by *An. minimus *s.l. (43.64%, n = 55), *An. annularis *(37.5%, n = 7), and *An. pallidus *(33.33%, n = 12) of the 17 collected species. The average HBI values for indoor and outdoor collections were 6.96% and 11.73%, respectively (Table 3). None of the hosts were identified for four species: *An. fluviatiles *James (n = 1), *An. jeyporiensis *James (n = 7), *An. tessellates *Theobald (n = 2), and *An. varuna *Iyengar (n = 2).

**Table 3:** Human blood index (HBI) of *Anopheles *species collected indoors and outdoors

Species	Indoor collections		Outdoor collections		Overall HBI
		
	N	HBI	N	HBI	
*An. aitkenii*	1	0			0
*An. annularis*	4	50	3	33.33	37.5
*An. baimai*	3	66.67	2	100	80
*An. barbirostris*	31	3.23	31	6.45	4.62
*An. hyrcanus *group	16	6.25	7	0	4.35
*An. jamesii*	2	50			33.33
*An. karwari*	41	2.44	16	6.25	3.39
*An. kochi*	1	0			0
*An. maculatus *s.l.	10	40	1	0	26.67
*An. minimus *s.l.	34	47.06	21	38.1	43.64
*An. nigerrimus*	28	0	1	0	0
*An. pallidus*	8	37.5	4	50	33.33
*An. peditaeniatus*	63	7.94	10	0	6.41
*An. philippinensis*	134	19.4	109	19.27	18.43
*An. subpictus*	27	14.81			12.12
*An. umbrosus*	24	0	81	6.17	4.55
*An. vagus*	550	10.55	55	25.45	10.29

Total	977	6.96	341	11.73	

### Multiple host feeding

Multiple host feeding was only detected in mosquitoes when they took blood from different types of hosts, which occurred in 74 individuals (5.61%). The observed combinations were human and goat (n = 37), human and bovine (n = 22), and human, bovine, and goat (n = 13). The highest number of multiple feedings was detected in *An. vagus *(62%, n = 46).

### Host preference

The forage ratio (*w_i_*), host selection index (*B_i_*), and G-test values for 1318 individuals (2456 tested) from 14 anopheline taxa are presented in Table 4. The bovine category was the most preferred animal, followed by goats/sheep and humans (Table 4). Relative preferences for humans occurred in *An. baimai *(*B_i _*= 0.61) and *An. minimus *s.l. (*B_i _*= 0.24). *Anopheles annularis*, *An. maculatus *s.l., and *An. pallidus *exhibited opportunistic blood-feeding behavior, in that they preferred to feed on accessible hosts, either human or animal. The remainder of species showed relative preferences for bovine hosts. For three species (*An. annularis*, *An. maculatus *s.l., and *An. pallidus*), the use of raw or log-transformed data to obtain G-test values for host abundance produced different results, whereas for the other 11 anopheline taxa, the two methods produced similar results in terms of preferred hosts (Table 4).

**Table 4:** Host selection index (*B*_*i*_) of *Anopheles *species in Kumari, Bangladesh

Species	Host data	Host selection index (*B_i_*)				G-test value	P-value
				
		Human	Bovine	Goat & Sheep	Others		
*An. annularis*	Raw	0.16	0.41*	0.43*	0	8.77	0.07
	Log-transformed	0.33*	0.39*	0.28*	0	7.63	0.11

*An. baimai*	Raw	0.61*	0.39*	0	0	6.10	0.19
	Log-transformed	0.77*	0.23*	0	0	9.62	0.05

*An. barbirostris*	Raw	0.02	0.89*	0.1	0	187.13	< 0.01
	Log-transformed	0.04	0.89*	0.07	0	153.70	< 0.01

*An. hyrcanus *group	Raw	0.02	0.91*	0.07	0	68.68	< 0.01
	Log-transformed	0.04	0.92*	0.05	0	57.06	< 0.01

*An. karwari*	Raw	0.01	0.92*	0.05	0.01	172.48	< 0.01
	Log-transformed	0.03	0.92*	0.04	0.01	143.38	< 0.01

*An. maculatus *s.l.	Raw	0.1	0.33*	0.53*	0.03	13.76	0.01
	Log-transformed	0.23*	0.34*	0.37*	0.06	9.35	0.05

*An. minimus *s.l.	Raw	0.22*	0.67*	0.11	0	67.85	< 0.01
	Log-transformed	0.39*	0.54*	0.06	0	73.23	< 0.01

*An. nigerrimus*	Raw	0	1.00*	0	0	108.45	< 0.01
	Log-transformed	0	1.00*	0	0	92.77	< 0.01

*An. pallidus*	Raw	0.15	0.56*	0.25*	0.04	11.90	0.02
	Log-transformed	0.29*	0.49*	0.15	0.06	11.26	0.02

*An. peditaeniatus*	Raw	0.03	0.89*	0.08	0.01	205.88	< 0.01
	Log-transformed	0.05	0.88*	0.06	0.01	169.71	< 0.01

*An. philippinensis*	Raw	0.08	0.84*	0.07	0.01	479.22	< 0.01
	Log-transformed	0.16	0.77*	0.04	0.02	415.25	< 0.01

*An. subpictus*	Raw	0.05	0.84*	0.05	0.05	60.31	< 0.01
	Log- transformed	0.1	0.78*	0.03	0.08	51.46	< 0.01

*An. umbrosus*	Raw	0.02	0.82*	0.17	0	297.72	< 0.01
	Log-transformed	0.04	0.84*	0.12	0	237.17	< 0.01

*An. vagus*	Raw	0.04	0.81*	0.12	0.03	1393.67	< 0.01
	Log-transformed	0.09	0.79*	0.08	0.05	1132.35	< 0.01

Threshold level for *B_i _*= 0.2; * indicates the preferred host.

Values of *B_i _*were not calculated for those species for which less than five individuals were captured.

## Discussion

The present study confirmed that anopheline species composition can vary with different sampling methods. Only four species (*An. philippinensis*, *An. minimus *s.l., *An. baimai*, and *An. annularis*) were collected using human bait (HB), the most vital method for discriminating anthropophilic species. *Anopheles baimai *and *An. annularis *were dominant only in HB collections. Light traps (LTs) captured all species and can be considered the most neutral method for collecting mosquitoes. However, the differences between indoor or outdoor LT catches indicate that attention must be paid to where LTs are set. Six species were collected using pyrethrum spray (PS); however, more than 93% were *An. vagus*. Mosquito species composition differed between LT and PS collection methods, and only *An. vagus *and *An. subpictus *were collected more often by PS. These results verified the effectiveness of HB, which only collects anthropophilic species, and demonstrated that the application of PS is not particularly useful for collecting anthropophilic species.

The prevalence of malaria is influenced by host preferences of *Anopheles *species, but little information is available on such preferences in Bangladesh [32]. In nature, the expression of host preference (selection of host) by a mosquito may depend on several extrinsic or intrinsic factors [33,34]. In the present study, the HBI values of mosquitoes collected indoors and outdoors were 6.96% and 11.73%, respectively. The higher HBI in outdoor-collected mosquitoes suggests two possibilities: people may be bitten more frequently outdoors, or indoor-biting mosquitoes do not remain inside and instead exit at night. Both possibilities may indicate that mosquitoes are becoming exophagic or that exophagic traits are favored by insecticidal pressure of bed nets. *Anopheles baimai*, an efficient malaria vector in Bangladesh, usually rests and bites humans outdoors, even though it is highly anthropophilic [6,35,36]. The higher HBI in outdoor-collected anophelines along with the higher outdoor-biting tendency of malaria vectors in Bangladesh [6,35] indicate that bed nets or other devices used indoors may not provide enough protection from vectors.

*Anopheles *species incriminated as malaria vectors exhibit preferences for humans [34]. Ramsay *et al. *[37] reported a substantial preference for human blood by *An. minimus*, which was supported in part by Toumanoff and Hu [38] in Vietnam. However, this species (collected outdoors) exhibited a low HBI in the Philippines [39] and India [40]. We found that both bovine (*B_i _*= 0.54) and human (*B_i _*= 0.39) hosts were preferred by *An. minimus *s.l. in Kumari, Bangladesh.

*Anopheles maculatus *has been reported as an important malaria vector in the Malay Peninsula but is considered less important in Bangladesh [6], Assam, Borneo [37,41], and the Philippines [39]. Wharton [42] reported that the Malayan *An. maculatus *feeds uniformly on animals, although it exhibits a slight preference for humans even when animals are accessible. We found that both humans (*B_i _*= 0.23) and bovines (*B_i _*= 0.34) were the preferred hosts of this species. Similar results were reported in Hong Kong [43], whereas *An. maculatus *appears to feed largely on bovine hosts in the Philippines [39]. Thus, *An. maculatus *feeds on either bovine or human hosts, whichever is more abundant or accessible.

A higher value of the HBI in an *Anopheles *species indicates that it can function as a malaria vector [36]. In our study, higher values of HBI were found in *An. baimai *(80%), *An. minimus *s.l. (43.64%), and *An. annularis *(37.50%). These three species have been recognized as malaria vectors in Bangladesh, and the former two species play a main role in transmitting malaria in hilly and forested areas [6,35,36]. Very low to negligible values of HBI were found in *An. kochi*, *An. nigerrimus*, *An. aitkenii*, *An. karwari*, the *An. hyrcanus *group, *An. umbrosus*, and *An. barbirostris*. Recently, Alam *et al. *[20] reported a high malaria infection rate for *An. karwari*, *An. barbirostris*, *An. nigerrimus*, and *An. subpictus *in Bangladesh. In contrast, these species are generally zoophilic and prefer to feed on bovine blood [44]. In the present study, we found low HBI in *An. karwari*, *An. barbirostris*, and *An. nigerrimus*. These species are considered to be non-vectors, with the exception of *An. subpictus*, on the Indian subcontinent [41], which is in agreement with our observed HBI values for these species. Some non-vector species may be overestimated as malaria vectors due to the methods used [20]. For example, using the entire mosquito body in ELISA can lead to over evaluation of vectors because *Plasmodium *species in human blood within mosquitoes are more likely to be detected. It is essential to check for *Plasmodium *within the upper parts of the mosquito body (thorax and head), and an even more reliable method is dissection because blood is often dispersed throughout the mosquito body and not only in the abdomen.

The densities of potential hosts in the study area must be measured to obtain a better understanding of mosquito host preferences [45]. The selection index (*B_i_*) enables the evaluation of mosquito host preferences with consideration of environmental conditions. A strongly anthropophilic mosquito species would only use humans as preferred hosts, whereas an opportunistic species would prefer more than one host. The value of *B_i _*for a particular mosquito species quantifies the intensity of preference, and the number of preferred hosts is a measure of the opportunistic behavior. *Anopheles minimus *s.l., *An. annularis*, *An. maculatus *s.l., and *An. pallidus *showed opportunistic blood-feeding behavior, indicating that they chose either human or bovine hosts depending on conditions. The other species exhibited clear preferences for bovine hosts. Therefore, the selection index (*B_i_*) results demonstrated that most of the mosquito species preferred bovines and goats as their hosts but not humans. An accurate host population estimation would be rather difficult for estimating the forage ratio. However, forage ratios are more powerful indicators for examining mosquito host blood-feeding preferences compared to other specialized indices [46]. These ratios may be used to compare the feeding preferences of various mosquito species in different areas.

*Anopheles annularis *and *An. vagus *are zoophilic, exophilic and exophagic in nature; however, they have been considered malaria vectors in India [41]. These two species were identified as vectors during epidemics in the floodplain areas of Bangladesh. They may have been implicated perhaps due to the low availability of nonhuman mammalian hosts [9,10]. Using gel-diffusion methods, a blood-meal analysis of several mosquito species in Dhaka, including three anophelines, indicated that *An. vagus *and *An. barbirostris *were highly zoophilic [32], which is in accordance with our results.

We identified a considerable proportion (5.61%) of multiple blood meals, a majority of which had been taken from humans and goats. We hypothesize that this combination was particularly frequent because goat shelters are, in many cases, located close to human sleeping rooms. The highest proportion of mixed blood meals occurred in *An. vagus*, which was collected indoors using PS. Multiple blood-feeding probably occurred due to disturbances or climatic factors [47]. Multiple blood-feeding is common during a single gonotrophic cycle among mosquitoes, and its epidemiological importance is controversial [47,48]. The loss of sporozoites by biting nonhuman animals during mixed feeding could be important in malaria control.

## Conclusions

We studied the blood-feeding patterns of *Anopheles *mosquitoes in a malaria-endemic area of Bangladesh using three sampling methods. We demonstrated that *An. baimai*, *An. minimus *s.l., *An. annularis*, *An. jamesii*, *An. maculatus *s.l., and *An. pallidus *are more or less anthropophilic, whereas most of the other species are zoophilic. The forage ratio (*w_i_*) and host selection index (*B_i_*) allows the prediction of future changes in mosquito host preference in accordance with the availability of host animal abundance. Mosquito species composition was methodology-specific, and LT sampling serves as a neutral reflection of the relative abundance of fauna. In contrast, human-bait and indoor spray catches are only composed of anthropophilic and endophagic species, respectively. Preliminary surveys using the proper sampling methods are crucial for identifying malaria vectors in Bangladesh.

## Competing interests

The authors declare that they have no competing interests.

## Authors' contributions

TN designed the study. KB, TN, and TUA conducted the fieldwork. KB performed the ELISA and PCR assay of mosquito blood meals. KB, TN, TUA, and AJH collaborated in writing the manuscript. All authors have read and approved the final manuscript.
